# Metabolic Depression in Cunner (*Tautogolabrus adspersus*) Is Influenced by Ontogeny, and Enhances Thermal Tolerance

**DOI:** 10.1371/journal.pone.0114765

**Published:** 2014-12-16

**Authors:** Nick I. Kelly, Abdullah Alzaid, Gordon W. Nash, A. Kurt Gamperl

**Affiliations:** Department of Ocean Sciences, Memorial University of Newfoundland, St. John's, Newfoundland and Labrador, Canada; Central Michigan University, United States of America

## Abstract

To examine the effect of ontogeny on metabolic depression in the cunner (*Tautogolabrus adspersus*), and to understand how ontogeny and the ability to metabolically depress influence this species' upper thermal tolerance: 1) the metabolic rate of 9°C-acclimated cunner of three size classes [0.2–0.5 g, young of the year (YOY); 3–6 g, small; and 80–120 g, large (adult)] was measured during a 2°C per day decrease in temperature; and 2) the metabolic response of the same three size classes of cunner to an acute thermal challenge [2°C h^−1^ from 10°C until Critical Thermal Maximum, CT_Max_] was examined, and compared to that of the Atlantic cod (*Gadus morhua*). The onset-temperature for metabolic depression in cunner increased with body size, i.e. from 5°C in YOY cunner to 7°C in adults. In contrast, the extent of metabolic depression was ∼80% (Q_10_ = ∼15) for YOY fish, ∼65% (Q_10_ = ∼8) for small fish and ∼55% (Q_10_ = ∼5) for adults, and this resulted in the metabolic scaling exponent (*b*) gradually increasing from 0.84 to 0.92 between 9°C to 1°C. All size classes of cunner had significantly (approximately 60%) lower routine metabolic rates at 10°C than Atlantic cod. However, there was no species' difference in the temperature-induced maximum metabolic rate, and this resulted in factorial metabolic scope values that were more than two-fold greater for cunner, and CT_Max_ values that were 6–9°C higher (∼21 vs. 28°C). These results: 1) show that ontogeny influences the temperature of initiation and the extent of metabolic depression in cunner, but not O_2_ consumption when in a hypometabolic state; and 2) suggest that the evolution of cold-induced metabolic depression in this northern wrasse species has not resulted in a trade-off with upper thermal tolerance, but instead, an enhancement of this species' metabolic plasticity.

## Introduction

Metabolic depression is a physiological strategy employed by species of all major animal phyla to maximize survival time in unfavourable environmental conditions such as extremes in temperature, hypoxia, desiccation, hypersalinity and food deprivation [1], [2]. This strategy relies on endogenous energy reserves, and typically involves a combination of a reduction in energy utilization (e.g. activity) and physiological functions (heart rate, breathing, or ventilation rate), and the downregulation or cessation of non-essential cellular functions such as anabolism and growth [1], [3], [4]. The extent of metabolic depression can vary from 80% of resting metabolic rate to a complete absence of measurable metabolism [1]–[3], [5], [6]. Although metabolic depression has been examined in various fish species, research has largely focused on its implications for surviving hypoxia/anoxia [4], [7], [8] or dessication [5], [9]. For example, lungfish, which aestivate to avoid desiccation, reduce oxygen consumption by 85%, and significantly decrease breathing, heart rate and blood pressure [5]. In contrast, only a few studies [10]–[13] have investigated the role of metabolic depression in fish species in response to low environmental temperatures.

The cunner (*Tautogolabrus adspersus* Walbaum) is a member of the family Labridae (wrasses) that inhabits inshore marine environments of the western Atlantic from Chesapeake Bay, USA to Newfoundland, Canada – their northern limit of distribution [14], [15]. During winter, the cunner exhibits dormancy (i.e. ceases activity and feeding) in response to the low environmental temperatures of its habitat [16], and this response is concomitant with a cold-induced, active, downregulation of metabolism. For example, [17] reported a decrease in routine metabolic rate in cunner from Woods Hole (Massachusetts, USA) when seawater temperatures declined from 12 to 6°C (temperature coefficient, Q_10_ = 8.50), and [13] reported acute and seasonal Q_10_ values for metabolic rate of 8–10, respectively, and a seasonal Q_10_ value for cardiac output of 7.9 between 5°C and 0°C for cunner from Newfoundland, Canada. When seasonal temperature fell from 8°C to 0°C, the protein synthesis in several tissues of Newfoundland cunner decreased by ≥55% [18]. Finally, [12] showed that the ability of cunner to depress their metabolism at cold temperatures (1°C) enhanced their hypoxia tolerance, and that metabolic depression in this species is dependent on other environmental variables such as water oxygen level and photoperiod. However, how the capacity for metabolic downregulation relates to the upper thermal tolerance and metabolic capacity of this species, and fishes in general, is not known. For example, it might be hypothesized that the evolution of physiological strategies/mechanisms that allow for metabolic depression at cold temperatures are at the expense of this species' ability deal with warm temperatures [i.e. there is a tradeoff between the capacity for metabolic depression (cold tolerance) and tolerance for warm temperatures]. Further, all the above studies have been conducted on large (adult fish), and it is possible that ontogeny (body size) influences the thermal physiology of cunner, including the extent (degree) of cold-induced metabolic depression, the temperature at which metabolic depression is initiated, and/or the relationship between metabolic downregulation and upper thermal tolerance.

In this study, we measured the oxygen consumption of three size classes of cunner [young of the year (YOY), ∼0.2–0.5 g; small, ∼3–6 g; and large (adults), ∼100 g] as water temperature was gradually lowered from 9 to 1°C. In addition, we examined the metabolic response of these same 3 size classes of cunner to an acute thermal challenge (2°C h^−1^) until loss of equilibrium (i.e. they reached their critical thermal maximum, CT_Max_), and compared these data to those of the same size classes of Atlantic cod (*Gadus morhua* Linneaus). As a North Atlantic teleost species that does not undergo metabolic depression, the Atlantic cod provides a useful comparison.

## Materials and Methods

The cunner used in this research were collected by the Ocean Sciences Centre's (OSC, Memorial University of Newfoundland) field services unit under the collection permit issued to it by the Department of Fisheries and Oceans (Canada). All experimental procedures described herein were approved by the Institutional Animal Care Committee of Memorial University of Newfoundland (Protocols 07-10-KG 09-16-KG) and performed in accordance with the guidelines of the Canadian Council on Animal Care.

### Experimental Animals

Wild cunner (*Tautogolabrus adspersus*) of different sizes (0.2 g–100 g) were collected using seines or hand nets in Portugal Cove, Newfoundland. These fish were held at the OSC for several months before they were separated into three experimental groups based on size (0.2–0.5 g, YOY; 3–6 g, small; and 80–120 g, large). Each experimental group was placed into a separate tank (smaller size classes in 20 L aquaria; the largest size class in a 1×1×0.5 m tank) supplied with circulating aerated seawater at ∼10°C and exposed to a 12 h light∶12 h dark photoperiod. Three times a week YOY cunner were fed a mixture of daphnia and brine shrimp; fish 3–6 g were fed a mixture of brine shrimp, mysid shrimp and chopped herring (*Clupea harengus*); and the largest fish (adults) were fed chopped herring. The fish used in the experiments investigating the extent and initiation of metabolic depression were later acclimated to 9±0.5°C and exposed to a natural fall/winter photoperiod (9 hours light: 15 hours dark) for at least 6 weeks. Fish were not fed for at least two days before measurements of metabolic rate or critical thermal maxima (CT_Max_) were made. Note: No fish was used in more than one experiment/procedure.

Atlantic cod (*Gadus morhua*) in the two lower size ranges specified for the cunner were obtained from the Joe Brown Aquatic Research Building (JBARB), Logy Bay, Newfoundland, where they were reared using standard protocols at 10–11°C. Due to the fact that cod in the large size-class were not available for experimentation, data on larger (∼70 g cod; Gamperl & Canada unpublished) and adult cod (∼1200 g; [19]) were used to examine the relationship between CT_Max_ and body mass for the cod. This data was collected on cod from the same founder population (i.e. reared at the OSC), and using similar equipment and initial water temperatures (8–12°C).

### Experiment ^#^1: Ontogeny of Metabolic Depression

All cunner were removed from their holding tanks 36–48 hours after their last feeding, blotted dry, weighed using an electronic scale, and transferred into one of three respirometers (see below) at 9°C. After a 48-hour recovery period from transfer and handling, resting oxygen consumption (MO_2_) was recorded for approximately 40 minutes. Following this, temperature was decreased by 1°C, a second measurement of MO_2_ was made approximately 7 hours later (i.e. MO_2_ was measured at approximately 9:00 AM and 4:00 PM), and the water temperature was reduced a further 1°C. This protocol was repeated for four additional days allowing for measurements on fish from 9 to 1°C. The 7 hour interval between measurements was sufficient for the MO_2_ of cunner to stabilize following a drop in temperature [13].

Oxygen consumption (in mg O_2_ kg^−1^ h^−1^) measurements were made on 7 to 9 individuals with flow velocity set to the minimal that would allow for effective mixing of the water (i.e. a velocity where the fish would rest calmly in the respirometers). MO_2_ measurements on fish less than 1 g and 3–6 g were made in 60 ml (custom made; Technical Services, MUN) and 200 ml (Loligo Systems, ApS, Tjele, Denmark) Blazka-type respirometers, respectively. Oxygen consumption of adult cunner (∼100 g) was measured in a 3.0 l (22×10×13.50 cm) rectangular, water jacketed, respirometer with a small internal mixing pump (Zoo Med Laboratories Inc. San Luis Obispo, CA, USA). These respirometers were supplied with aerated, temperature-controlled, and filtered seawater from a reservoir at a rate that ensured the water in the respirometers was fully saturated. To minimize visual disturbance, black plastic was draped over the respirometers and the behaviour of the fish was monitored during measurements via a mirror placed behind the respirometers

### Experiment^ #^2: Metabolic Capacity and Upper Thermal Tolerance

In these experiments, temperature was increased by 2°C h^−1^, and the temperature at which cunner and cod lost equilibrium was used as the measure of CT_Max_ [19]. All fish were food deprived for at least 16 hours before collection, and allowed to recover from handling/transport in the respirometer overnight (i.e. for approximately another 16 hours). This minimized the effects of specific dynamic action (SDA) and stress on the fish's metabolic rate. Oxygen consumption measurements were made at 10°C (acclimation temperature) and during the last 15 minutes at each 2°C temperature interval. Preliminary experiments showed that temperature had reached a steady state by this time. This protocol (45 minute temperature increase, followed by 15 minutes at stable temperature) was continued until the fish lost equilibrium. MO_2_ measurements were not taken after the fish lost equilibrium, as water temperature was gradually (i.e. over approximately 30 min. to 1 h) reduced to 10°C in an effort to recover all of the fishes. However, a small proportion (<10%) did succumb to the experimental protocol. The day after the CT_Max_ of each fish was determined, the fish were removed from the respirometer, blotted dry, weighed using an electronic scale accurate to 0.001 g, and returned to their holding tank.

The same respirometers, as described in Experiment #1, were used to measure the fish's MO_2_ during the CT_Max_ experiments. However, during respirometry measurements on Atlantic cod, the YOY size class of this species was quite active in the respirometer. To address the potential effects of this behavior on the fish's CT_Max_ – temperature relationship and CT_Max_, an additional thermal tolerance experiment was conducted for both species. Briefly, 9–12 cunner or cod were placed in a 22×12×17 cm, sealed, rectangular temperature controlled tank where they were free-swimming and could interact with conspecifics. Temperature was increased using the same protocol as for the respirometer experiments (2°C increase hour^−1^, starting at 10°C and continuing until the CT_Max_ was reached). The CT_Max_ of each fish was recorded as they lost equilibrium. CT_Max_ values for free-swimming YOY cod and cunner were compared to values obtained using the 60 ml respirometers. Note: The large volume of the tank (respirometer) compared to the mass of the fish precluded the accurate measurement of RMR as temperature was increased.

### Oxygen Consumption Measurements

Oxygen consumption (MO_2_) was measured by turning off the inflow of water to each respirometer, and measuring the drop in water oxygen content using a Fiber-Optic oxygen meter (FIBOX 3 LCD, PreSens, Germany) and a pre-calibrated dipping probe that was inserted into the respirometer. Data from the O_2_ meter (i.e. water oxygen content in mg l^−1^) was directly recorded to a computer running OxyView software (PreSens, Germany; PST3 V5.32 for the 60 ml respirometer; and LCDPST3 V1.16 for the 200 ml, and 3 l respirometers). The data from OxyView was then downloaded into Logger Pro (Version 3.4, Vernier Software and Technology, Beaverton, OR, USA), and the rate of decline in water O_2_ content (in mg O_2_ l^−1^ min^−1^) was determined by fitting a linear regression to the O_2_-time data. Oxygen consumption (MO_2_) was then calculated using the formula:
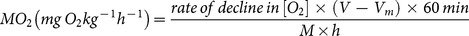
where V is respirometer volume in liters; V_m_ =  the mass (volume) of the fish, assuming that 1 g is equal to 1 ml of seawater; M =  mass of the fish in kg; h =  hour, and [O_2_] is water oxygen content in mg l^−1^, corrected for the lower solubility of oxygen in seawater vs. freshwater (there is no salinity correction in the OxyView software).

During thermal tolerance experiments, maximum oxygen consumption (MMR) for each fish was the MO_2_ value obtained just prior to the fish losing equilibrium, absolute metabolic scope (AMS) was calculated as maximum MO_2_ – the fish's MO_2_ at 10°C, and factorial metabolic scope (FMS) was calculated as maximum MO_2/_MO_2_ at 10°C. Mean Q_10_ values for each species-size combination were calculated using the formula:
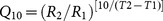



Where: R_1_ =  Metabolic rate at 10°C (T_1_) and R_2_ =  Metabolic rate at the temperature the fish lost equilibrium (T_2_).

**Figure 1:**
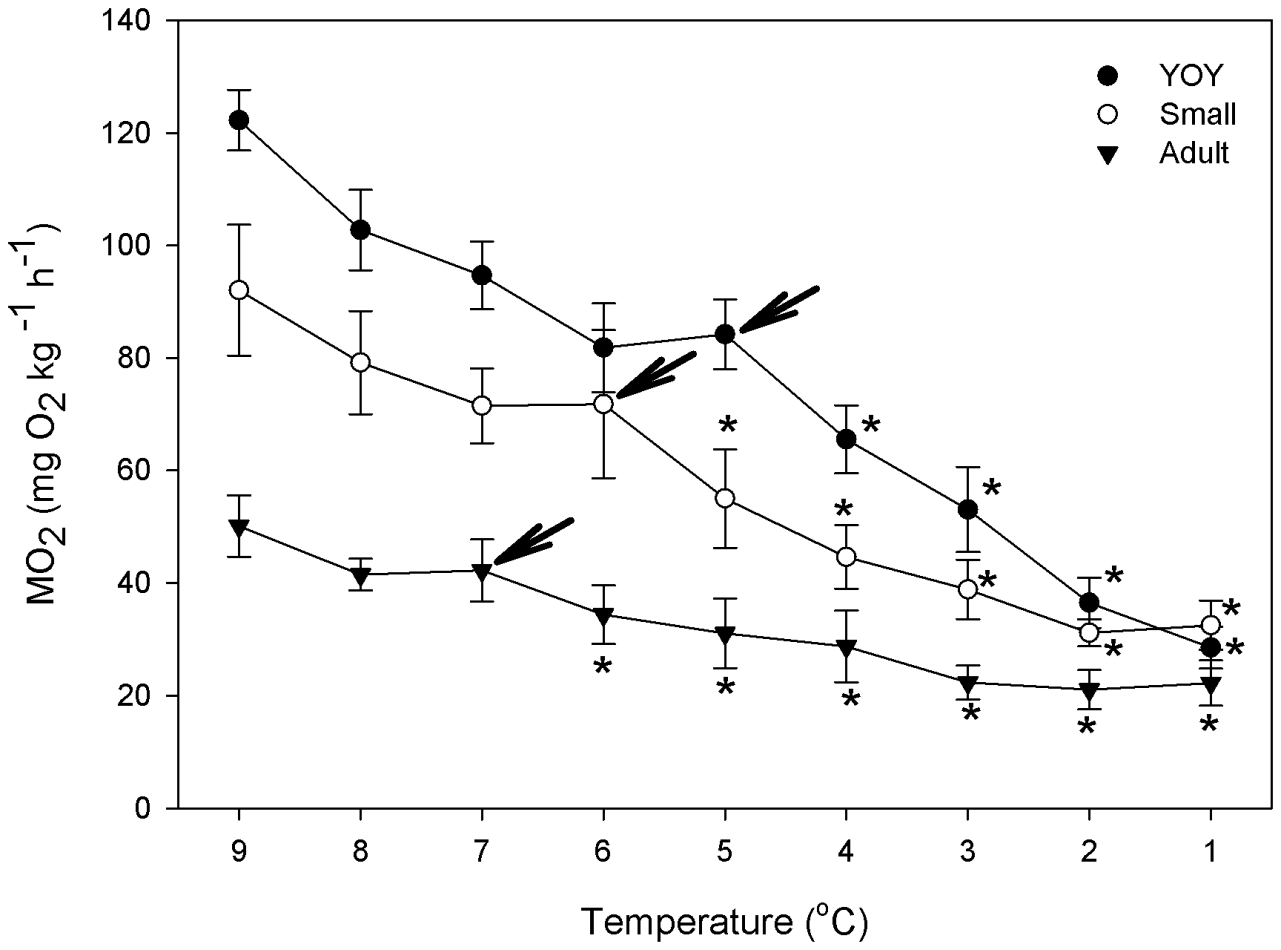
The effect of decreasing temperature at 2°C day^−1^ on oxygen consumption (MO_2_) of young-of-the-year (YOY), small and adult (large) cunner. A repeated measures one-way ANOVA, followed by Dunnett's post-hoc tests (P<0.05), was used to identify significant differences (*) in MO_2_ between the acclimation temperature (9°C) and the other temperatures within each size class. The arrows indicate the temperature at which a rapid decrease in MO_2_ was initiated for each size class. Values are mean ±1 S.E. (N = 7 - 8 for each size class).

In addition, Q_10_ values for changes in cunner MO_2_ with decreasing temperature were calculated over several temperature ranges using the mean MO_2_ data for all groups: 1) from 9°C to 1°C (Overall Q_10_); 2) from 9°C until MO_2_ began decreasing at a much faster rate (Q_10_ before), this point taken as the temperature where metabolic depression was initiated; and 3) from the temperature at which metabolic depression was initiated to the temperature at which MO_2_ stopped decreasing (Q_10_ after) (see Table 1 and Fig. 1).

**Table 1:** Morphometric and metabolic parameters for 9°C acclimated cunner of various size classes [YOY, young-of-the-year; small and large (adult)] when exposed to a temperature decrease from 9°C to 1°C at 2°C day^−1^.

	Size (Age) Class		
	YOY	Small	Large
**N**	8	8	7
**Mass (g)**	0.30±0.04^a^	4.24±0.31^b^	113.49±5.76^c^
**MO_2_ at 9°C**	122.25±5.40^a^	92.07±11.64^b^	50.13±5.46^c^
**MO_2_ at 1°C**	28.54±3.71^a^	32.50±4.36^a^	22.24±4.01^a^
**Overall Q_10_ (9**–**1°C)**	6.16	3.68	2.76
**Q_10_ Before**	2.54 (9–5°C)	2.29 (9–6°C)	2.35 (9–7°C)
**Q_10_ After**	14.96 (5–1°C)	8.05 (6–2°C)	4.92 (7–3°C)

Within each row, values without a letter in common are statistically different (P <0.05). Values in brackets indicate the temperature ranges over which the presented Q_10_ values were calculated. Values are mean ± 1 S.E.

The respirometers were cleaned twice weekly with 70% ethanol to minimize background bacterial contamination. In addition, blank measurements were performed after each MO_2_ measurement to quantify any possible bacterial oxygen consumption. In the majority of cases there was negligible background oxygen consumption. However, in cases where a background rate of oxygen consumption was observed, this value was subtracted from the data for individual fish. Because of the inactive nature of cunner, oxygen consumption measurements were assumed to approximate the fish's resting/routine metabolic rate (RMR).

### Data and Statistical Analyses

#### Experiment #1

The temperature at which metabolic depression was initiated and the extent (degree) of metabolic depression were determined for each group from the MO_2_ - temperature data. The temperature at which metabolic depression was initiated was taken as the temperature at which there was a noticeable change in the slope of the MO_2_ - temperature relationship for each group. Arrhenius plots of the data were also made for all groups. However, this analysis did not substantially improve the interpretation of the data.

Initially, a repeated measures two-way ANOVA was used to examine the effects of size class and temperature on the RMR of cunner in this experiment. However, this analysis revealed a significant interaction between the two main effects. Thus, further statistical analyses were restricted to: 1) one-way ANOVAs at 9°C and 1°C to test whether RMR was different between size classes at these temperatures; and 2) repeated measures one-way ANOVAs followed by Dunnett's post-hoc tests to identify significant differences in RMR between the acclimation temperature (9°C) and the other temperatures for each size class. Log_10_ RMR was plotted against log_10_ wet mass at each temperature during the experiments examining the effect of size/age on metabolic depression, and mass scaling exponents (*b*) were calculated as the slope of these relationships +1.

#### Experiment #2

The main effects of species and size (age) on RMR, MMR, AMS, FMS, Q_10_ values and CT_Max_ were initially examined using a two-way ANOVA. When the main effect of body size for a particular parameter was significant, a one-way ANOVA followed by t-tests was used to identify significant differences between the cod (only 2 size/age classes used), while one-way ANOVAs followed by Tukey's post-hoc tests were used for the cunner. When the main effect of species was significant, t-tests were performed within each size class. Finally, one-way ANOVA's were used to determine if CT_Max_ values measured using the respirometer were significantly different from those obtained for YOY fish tested in the ‘tank’ environment. Linear regression analysis was used to determine the slope of the relationship between log_10_ body mass and the log_10_ of the various metabolic parameters, for each species, during the CT_Max_ experiment, including: RMR, MMR, FMS and AMS. Mass scaling exponents (b) were calculated as above.

All statistical tests were performed using GraphPad Prism (GraphPad Prism version 5.0b for Mac, GraphPad Software, San Diego California USA) and P<0.05 was used as the level of statistical significance unless otherwise stated. All values presented in the text, figures and tables are means ±1 standard error of the mean (S.E.).

## Results

### Experiment ^#^ 1: Ontogeny of Metabolic Depression

At the acclimation temperature of 9°C, the RMR of the three size classes of cunner was significantly (P<0.05) different (Table 1), with the RMR value of YOY cunner being 1.33 and 2.44 fold greater as compared with small and adult fish, respectively. This difference in MO_2_ values between the three size classes resulted in a mass exponent (*b*) of approximately 0.84 at this temperature. For all three size classes, a decrease in temperature from 9°C to 1°C resulted in a dramatic drop in metabolic rate to a level where there was no significant difference in MO_2_ between the three size classes (Fig. 1). The Q_10_ values for the overall drop in metabolic rate between 9°C and 1°C were 2.76, 3.68 and 6.16 for adult, small and YOY cunner, respectively (Table 1).

However, the relationship between decreasing temperature and RMR was not linear, and could be divided into three distinct phases: 1) a gradual decrease in RMR where Q_10_ values ranged between 2.29 and 2.54 for all the three size classes; 2) a phase characterized by a more rapid decrease in RMR until each size class reached a minimal value; and 3) a final phase (at least for adult and small cunner) where RMR was temperature insensitive. Importantly, although the rapid drop in RMR occurred over a 4 degree range for all size classes, it was initiated at different temperatures (7°C, 6°C and 5°C for adult, small and YOY cunner, respectively), and the drop in MO_2_ over this temperature range was much greater for YOY cunner (Q_10_ = 14.96) as compared with small (Q_10_ = 8.05) and large (Q_10_ = 4.92) fish (Table 1).

**Figure 2:**
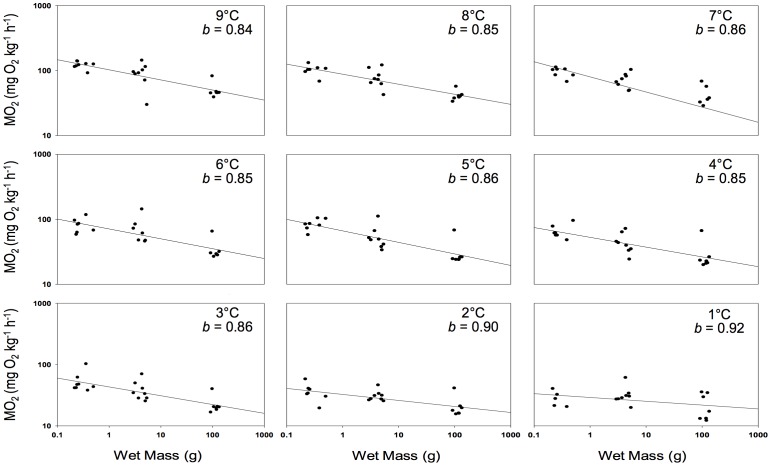
The relationship between body mass and oxygen consumption (MO_2_) for young-of-the-year (YOY), small and large (adult) cunner during an 8°C temperature decrease over 4 days. The mass scaling exponent (*b*) at each temperature was calculated as the slope of the log – log relationship plus 1.

As temperature was initially lowered, there was little change in the mass exponent for MO_2_ and it ranged from 0.84 to 0.86. However, because the metabolic rate of adult and small fish did not decrease further after 3°C and 2°C respectively, this resulted in *b* values of 0.90 and 0.92 at 2°C and 1°C, respectively (Fig. 2), and RMR values that were not significantly different at 1°C (Table 1).

### Experiment #2

#### Metabolic Parameters and Thermal Tolerance

**Figure 3:**
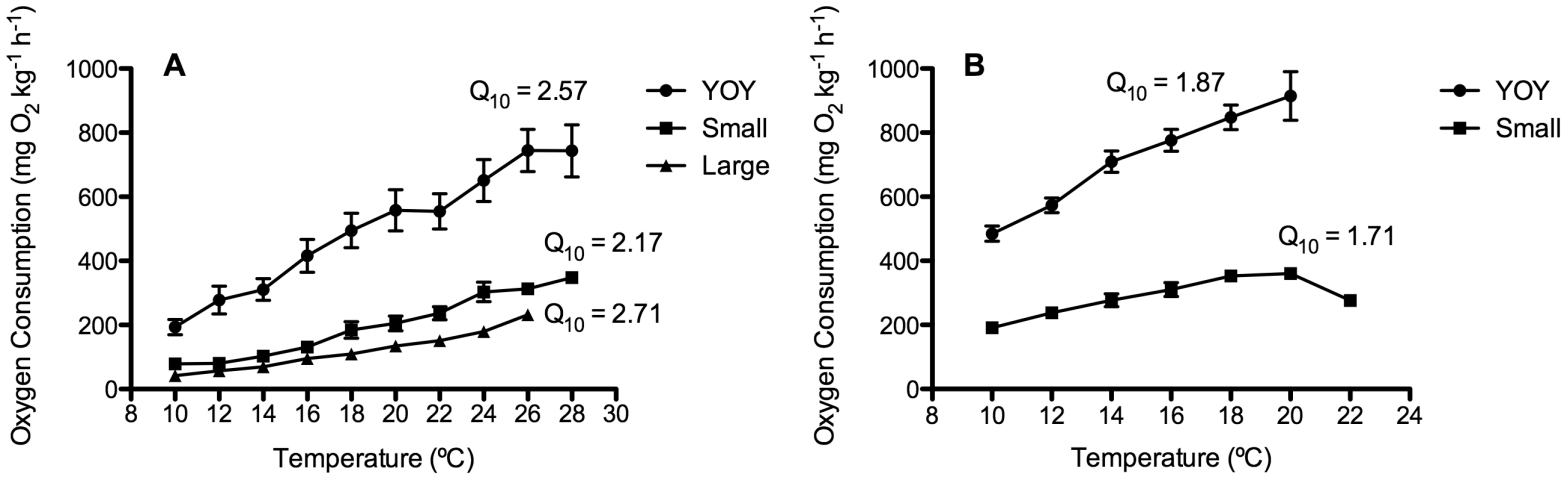
The effect of increasing temperature at 2°C h^−1^ until loss of equilibrium (CT_Max_) on oxygen consumption in (A) cunner and (B) Atlantic cod. Each line represents one size class: YOY (0.2–0.5 g), small (3–6 g), or large (∼100 g). Values are means ±1 S.E.

**Figure 4:**
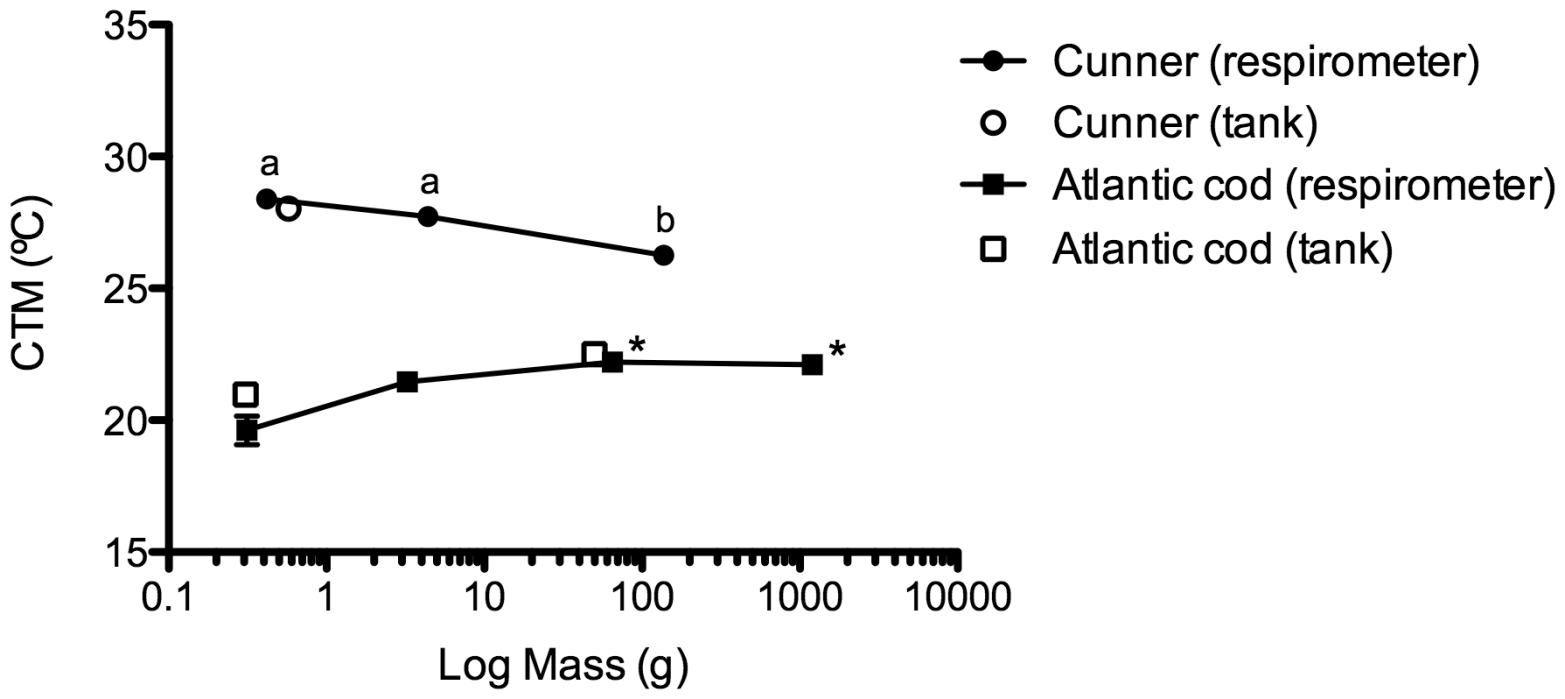
The relationship between body mass and CT_Max_ in cunner and Atlantic cod. An asterisk (*) indicates data plotted from previous studies ([22]; Gamperl and Canada, unpublished), whereas dissimilar letters indicate a significant difference (P<0.05) in CT_Max_ values between the three size classes of cunner. Note: the CT_Max_ values for YOY Atlantic cod (∼0.3 g) tested in the respirometer and the tank were significantly different (P<0.05). Values are mean ± 1 S.E.

For both species, and over the size ranges measured, oxygen consumption (MO_2_; mg O_2_ kg^−1^ h^−1^) increased with temperature but often leveled off at temperatures approaching the fish's CT_Max_ (Fig. 3). When comparing individuals of similar size between species, routine oxygen consumption rates were always higher for Atlantic cod than for cunner (Fig. 3; Table 2). For example, at 10°C the mean MO_2_ for YOY Atlantic cod (484.8±23.9 mg O_2_ kg^−1^ h^−1^, n = 7) was over 2-fold greater than for YOY cunner (193.3±24.0 mg O_2_ kg^−1^ h^−1^, n = 9). In contrast, the rate of increase in MO_2_ with temperature was slightly higher for the cunner (Q_10_ values for small and YOY cunner ranging from 2.17 to 2.60 as compared to 1.71 and 1.87 for cod, respectively; Table 2) and there was no difference in MMR between species. Given the lower RMR, but equivalent MMR, in cunner vs. cod it was not surprising to find that the AMS and FMS of YOY and small cunner were significantly greater than measured for the two size classes of cod (e.g. FMS values were 4.8 and 3.9 vs. 1.8 and 1.8 for cunner and Atlantic cod, respectively). In agreement with the difference in AMS and FMS between species, CT_Max_ values were approximately 6–9°C greater in cunner than cod (Fig. 4; Table 2). Values for cunner ranged from 26.3 to 28.4°C for the three size-classes, but were only 19.6°C±0.5 and 21.3°C±0.3 for YOY and small cod, respectively.

**Table 2:** Metabolic parameters and Critical Thermal Maximum (CT_Max_) values for 10°C acclimated cunner and Atlantic cod of various size classes [YOY, young of the year; small and large (adult)] when exposed to an acute temperature increase of 2°C h^−1^.

		Cunner	Atlantic Cod
**YOY** (∼ 0.2–0.5 g)	RMR	193.3±24.0^a+^	484.8±23.9^a^
	MMR	770.5±64.9^a^	874.1±36.5^a^
	AMS	577.1±69.4^a+^	389.3±27.9^a^
	FMS	4.8±0.9^a+^	1.8±0.1^a^
	Q_10_	2.6±0.3^a*^	1.9±0.1^a^
	CT_Max_ (°C)	28.4±0.3^a+^	19.6±0.5^a^
**Small** (∼ 3–6 g)	RMR	78.9±4.4^b+^	191.4±4.1^b^
	MMR	309.8±15.3^b^	345.1±19.3^b^
	AMS	220.3±20.4^b*^	158.7±19.4^b^
	FMS	3.9±0.4^a+^	1.8±0.1^a^
	Q_10_	2.2±0.1^a+^	1.7±0.1^a^
	CT_Max_ (°C)	27.7±0.2^a+^	21.3±0.3^b^
**Large** (∼ 100 g)	RMR	42.3±3.3^b^	
	MMR	208.9±12.9^b^	
	AMS	166.6±13.0^b^	
	FMS	5.1±0.5^a^	
	Q_10_	2.7±0.2^a^	
	CT_Max_ (°C)	26.3±0.2^b^	

Within each column, values without a letter in common are statistically different (P <0.05) between size classes. ^+^ and * indicate significant differences between the two species for a particular metabolic parameter at P <0.05 and P <0.06, respectively. Values are mean ± 1 S.E., N  =  7–10. RMR  =  routine metabolic rate. MMR  =  maximum metabolic rate. AMS and FMS  =  absolute and factorial metabolic scope, respectively.

#### Ontogeny of Thermal Tolerance and Metabolic Rate

**Figure 5:**
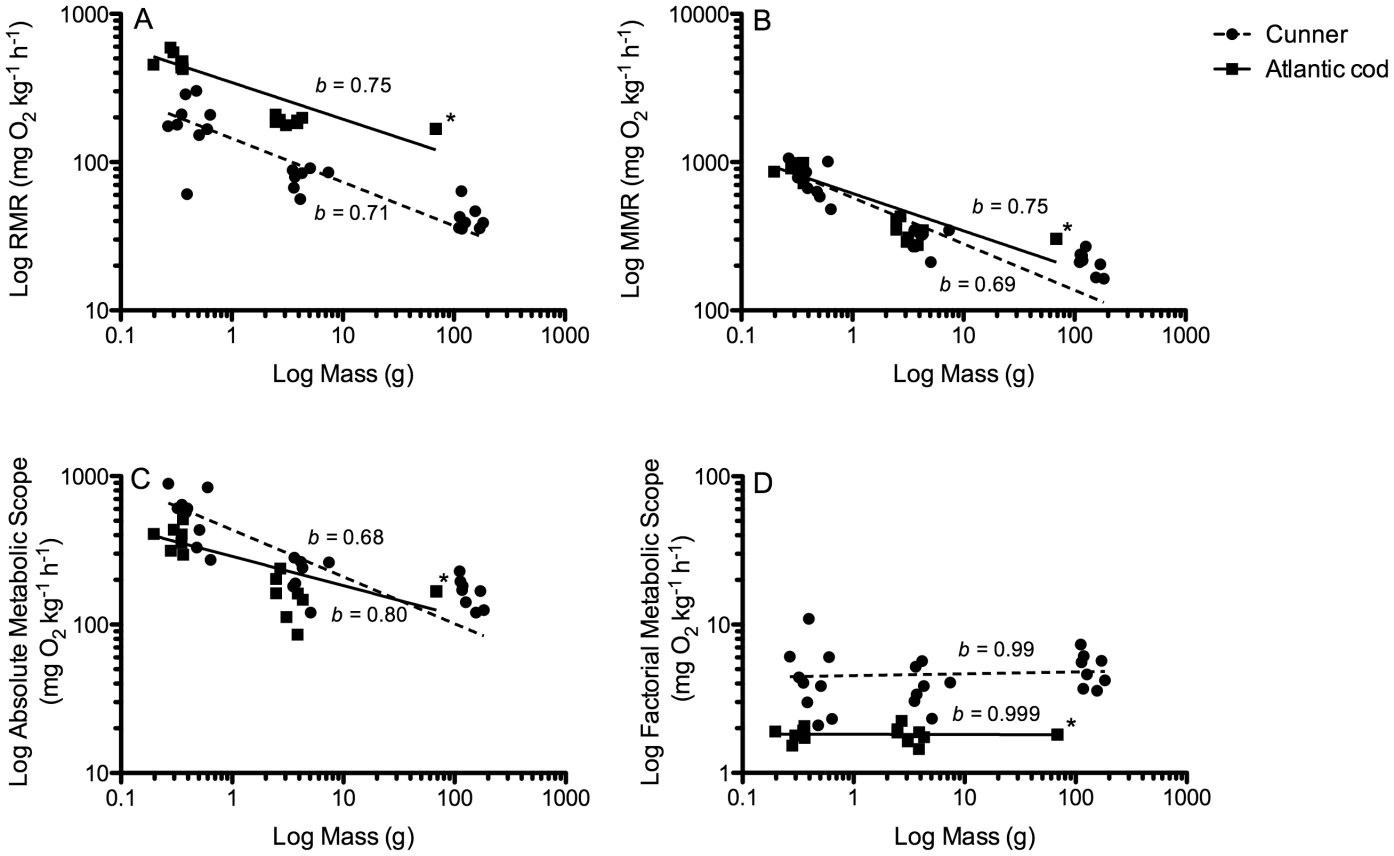
The effect of body size on metabolic parameters in cunner and Atlantic cod [Routine Metabolic Rate (RMR, A), Maximum Metabolic Rate (MMR, B), Absolute Metabolic Scope (AMS, C), and Factorial Metabolic Scope (FMS, D)]. Dashed and solid lines indicate linear regressions for the cunner and Atlantic cod, respectively. The mass scaling exponent (*b*) at each temperature was calculated as the slope of the log – log relationships plus 1. An asterisk (*) indicates an estimated value based on data from 9 Atlantic cod acclimated to, and tested at, both 8°C and 12°C (Gamperl and Canada, unpublished). All regressions were significant at p<0.001.

Oxygen consumption (in mg O_2_ kg^−1^ h^−1^) was highest in the YOY fish and decreased with size in both cunner and Atlantic cod (i.e. YOY> small> large). For example, from the smallest size class measured to the largest, RMR values ranged from 193.3±24.0 to 42.3±3.3 for cunner, and 484.8±23.9 to 191.4±4.1 for Atlantic cod (Table 2, Fig. 3). When the metabolic data were presented as a log-log plot, this resulted in distinctly negative relationships for RMR, MMR, and AMS (Fig. 5). The slopes of these relationships ranged from −0.197 to −0.316 (i.e. the mass scaling exponent (*b*) ranged from 0.803 to 0.684). In contrast, factorial metabolic scope was not affected by body size.

CT_Max_ decreased with increasing body mass in cunner, with values for YOY, small and large cunner of 28.4±0.3°C, 27.7±0.2°C and 26.3±0.2°C, respectively. However, only the CT_Max_ value for large cunner was significantly different from that of the other two size classes (Fig. 4). The relationship between body mass and CT_Max_ in Atlantic cod was different from that observed for cunner. For example, CT_Max_ values were 19.6°C±0.5 and 21.3±0.3 for YOY and small cod, respectively, while values determined in previous experiments for large (∼70 g) and adult (∼ 1200 g) fish from JBARB stocks were both approximately 22°C (Gamperl & Canada unpublished; [19]). However, this difference appears to be largely due to the behaviour of YOY cod in the respirometer. The CT_Max_ value for YOY cod that were free-swimming in the tank was significantly higher (by 1.4°C) as compared with those tested in the Blazka-type respirometer (Fig. 4). Collectively, this data suggests that the CT_Max_ of cod does not increase with body mass, or if it does, it increases only slightly (by ∼ 1.5°C).

## Discussion

### Ontogeny of Metabolic Depression

RMR decreased in all the three size classes of cunner as temperature was lowered. However, the magnitude of the decrease was dependent on the temperature range over which the change in RMR was measured (see Table 1). In the higher temperature range (between 9 to 7°C for adult, 9 to 6°C for small, and 9 to 5°C for YOY fish), RMR gradually decreased (Q_10_ = 2.35, 2.29, and 2.54 for adult, small and YOY fish, respectively) with water temperature (Fig. 1 and Table 1). These data are in agreement with the Q_10_ values reported by [17] for cunner from Woods Hole in the higher temperature range of her study (Q_10_ = 2.40; temperature 12–22°C), the data of [13] (Q_10_ = 2.50) for adult cunner from Newfoundland when exposed to a temperature drop from 14 to 5°C, as well as with [20] who reported a Q_10_ of 2.71 for the goldsinny wrasse (*Ctenolabrus rupestris*) between 10 and 6°C. These Q_10_ values are also within the general range (1.8–4) reported for the direct effect of temperature on chemical and biological processes [21] and on the resting/routine metabolic rate of other teleost fishes [2], [22], [23], [21], [24]. Thus, the drop in MO_2_ at the higher temperatures in this study can be considered a normal effect of temperature on metabolism.

In contrast, further reductions in temperature resulted in a more rapid decrease in the RMR for all the three size classes of cunner. Adult cunner showed a 55% decrease in RMR (Q_10_ = ∼5) between 7 and 3°C, the RMR of small cunner decreased by 65% (Q_10_ = ∼8) between 6 and 2°C, and YOY reduced RMR by 80% (Q_10_ = ∼15) between 5 and 1°C (Fig. 1 and Table 1). This abrupt drop in RMR over these 4°C temperature ranges was associated with very high Q_10_ values, and clearly indicates that the decrease in cunner RMR was related to an active downregulation of metabolism. Cold-induced metabolic depression, and its associated high Q_10_ values for metabolism, have been documented in several previous studies on this Northern wrasse species. For example, [13] showed that seasonal and acute temperature decreases from 5 to 0°C resulted in a 60–70% decrease in the RMR of adult Newfoundland cunner (Q_10_ = ∼10). Cunner from Woods Hole were reported by [17] to have high Q_10_ values for RMR when seawater temperature dropped from 12 to 6.4°C (Q_10_ = 5.50–8.90); the higher temperature (12°C) at which Woods Hole cunner initiated metabolic depression likely explained by the fact that this population is exposed to a warmer seasonal thermal range (2.5 to 22°C) than in Newfoundland (−1 to 14°C). Finally, the goldsinny wrasse, exhibits winter dormancy and dramatically decreases mean oxygen uptake rates (Q_10_ = 542.01) when temperature is lowered from 6 to 4°C [20]. Further evidence of the capacity of cunner to enter metabolic depression at similar temperatures to those reported in the present study comes from studies that have examined the cellular basis for this phenomenon in this species. For example, [18] measured the rate of protein synthesis in several tissues of cunner from Newfoundland, and reported that this species showed a large reduction (55–66%) in protein synthesis when the seawater temperature dropped from 8 to 4°C (Q_10_ values ranging from 6 to 21). Further, Gamperl, MacSween, & Staples (unpublished) have shown that indices of mitochondrial function (e.g. State 3 respiration) are much lower at 0°C than at 10°C (Q_10_ = ∼7–10).

Although RMR decreased across most of the temperature range to which the fish were exposed, it did not decrease at the lowest two temperatures for both large (adult) and small cunner (Fig. 1). This suggests that when the metabolism of this species is fully depressed there is no further scope for temperature-dependent decreases in RMR. Although this hypothesis needs to be examined further, our results are consistent with [17]. This author showed that although cunner from Woods Hole decreased their metabolic rate significantly from 12–6°C (Q_10_ = 8.50), no further decreases in RMR were recorded as temperature was lowered from 6 to 3°C.

While the evidence for cold-induced metabolic depression in cunner is convincing [12], [13], [17], [18], [25], [Gamperl, MacSween & Staples unpublished], it was not known prior to this study whether ontogeny affected aspects of metabolic depression in this or other fish species. Based on their greater mass-specific RMR than large fish at higher temperatures, and the limited availability of food for cunner during the winter months, it was predicted that YOY cunner would: 1) initiate metabolic depression at a higher temperature (earlier) than large fish; and/or 2) depress metabolism to a greater extent than larger (older) fish. It is clear from the RMR and Q_10_ data, where the latter were ∼15, 8 and 5 for YOY, small and adult cunner, respectively, that YOY cunner do indeed depress their metabolism to a greater extent than larger/older cunner when faced with cold temperatures. However, the data also reveal that the temperature at which metabolic depression is initiated in cunner increases with body mass [from 5°C for YOY cunner to 6°C and 7°C in small and large (adult) fish, respectively) (Fig. 1). This agrees with the anecdotal observations of [26] and [27] who suggested that large cunner become dormant from 1 to 3 weeks before smaller fish and emerge earlier. It is possible that, in contrast to older fish, YOY cunner do not have sufficient energy stores to sustain themselves over the winter and must feed as long as possible in the fall before metabolic depression is initiated. This hypothesis is supported by research on the bluegill (*Lepomis macrochirus*), which showed that larger fish emerge from the winter starvation period in better energetic condition (i.e. with a greater amount of stored lipids) than smaller individuals [28]. The authors attributed this positive relationship between body size and energy stores to the inverse relationship between body size and mass-specific metabolic rate.

The later initiation of metabolic depression, the greater decrease in RMR in YOY fish, and the temperature-insensitivity of RMR in small and large cunner at cold temperatures had an unexpected consequence. These 3 effects caused the scaling exponent (*b*) for RMR to increase from the 0.84–0.86 at 9–3°C to 0.90 at 2°C and 0.92 at 1°C (Fig. 2). This result provides further evidence against the use of a single, universal, value of *b* with regards to fish metabolism. Further, it shows that the scaling of metabolic rate with body size (age) in cunner is dependent on temperature. This finding is in agreement with the inverse relationship found between temperature and scaling exponents when the combined data of 89 species of teleost were examined [23].

### Metabolic Depression and Thermal Biology

The RMR of cunner (at 10°C) was approximately 60% lower than measured for the Atlantic cod. This result agrees with [25] who found that the routine metabolic rate of cunner was approximately 50% lower than that of other North Atlantic teleost species at temperatures ranging from 10 to 15°C. The lower RMR of cunner, combined with the similar MMR values between the two species, resulted in cunner having greater values for FMS and AMS as compared with Atlantic cod (Table 2; Fig. 5C and 5D). Further, since thermal tolerance is largely determined by a fish's cardiorespiratory capacity [29], [30], cunner had a CT_Max_ approximately 6–9°C greater than for the cod. Similar to this study, [12] also report significantly lower RMR values for cunner as compared to another North Atlantic teleost species, the Greenland cod (*Gadus ogac*), and that the low metabolic rate of cunner in comparison to Greenland cod provided an advantage with regards to surviving hypoxic conditions. Together, these results suggest that the lower resting/routine metabolic rate and larger metabolic scope of cunner makes them more tolerant of adverse environmental conditions as compared with other North Atlantic teleost species.

In the present study, metabolic rate, measured as oxygen consumption (MO_2_; mg O_2_ kg^−1^ h^−1^), increased with temperature for both species and all size classes (Fig. 1). The Q_10_ values for Atlantic cod in this study (1.7 and 1.9) are nearly identical to those reported by [31] (1.9) and Gamperl & Canada (unpublished) (1.7), but slightly less than those reported by [32] (2.2) or [19] for larger instrumented cod (2.7). Measurements of metabolic rate in cunner exposed to increasing temperatures have not been made previously. However, while the Q_10_ values of cunner obtained in this study (2.17–2.71) are slightly higher than measured for the Atlantic cod, they are within the range of those reported for other fish species (∼2–3: e.g. redband trout, [33]; and sockeye salmon, [34]). This suggests that the cunner's lower metabolic rate and capacity to downregulate their metabolism when exposed to cold temperatures does not affect the overall temperature sensitivity of physiological processes when they are not in metabolic depression.

### Ontogenetic Effects on Thermal Tolerance and Metabolic Rate

The influence of body mass (ontogenetic stage) on CT_Max_ was different between the cunner and Atlantic cod. The CT_Max_ of cunner decreased slightly as body mass increased, with the CT_Max_ of large cunner being significantly lower than that measured for both YOY and small cunner [maximum difference over the size range ∼2.0°C (Fig. 4, Table 2)]. In contrast, the CT_Max_ of Atlantic cod increased slightly as body mass increased (Table 2, Fig. 4), with the CT_Max_ of YOY cod being significantly lower than that measured for two larger size classes (approximately 1.7°C difference). However, the CT_Max_ of YOY cod in a relatively ‘unstressed’ condition (tank environment) was 1.4°C higher than for YOY cod measured in the respirometer (Fig. 4), and this suggests that there is little overall variation in CT_Max_ with body size for this species. Why the direction of the relationship between CT_Max_ and body mass differs between the two species is not known. However, we suspect that it may be related to size-related differences in the capacity of large vs. small cunner to depress their metabolism (see below).

The finding that CT_Max_ varies very little across a wide range of body masses agrees with the majority of studies that have been performed to date. For example, [33] found no correlation between body size and CT_Max_ for redband trout (*Oncorhynchus mykiss* gairdneri) over a 35-fold increase in size (40 g–1400 g). [35] studied the relationship between CT_Max_ and body size in seven species of reef-fishes, and only found small size-related differences. [36] showed that neither length nor condition influenced the critical temperature maximum of wild and domestic strains of brown trout (*Salmo trutta*) and rainbow trout (*Oncorhynchus mykiss*). [37] reported no significant effect of thermal tolerance between fry and adults for Nile tilapia (*Oreochromis niloticus*), channel catfish (*Ictalurus punctatus*), rainbow trout (*Oncorhynchus mykiss*) or largemouth bass (*Micropterus salmoides*), and only a 1.3°C or less difference for Rio Grande cutthroat trout (*Oncorhynchus clarkia virginalis*) and apache trout (*Oncorhynchus gilae apache*). Finally, although several other authors have reported that the temperature tolerance of fishes varies with size (age) [38]–[41], variation in this parameter is usually 2°C or less.

For both Atlantic cod and cunner, the log-log relationships between RMR, MMR and AMS, and body mass, had a negative slope (Fig. 5); i.e. mass-specific metabolism decreased with body size. The negative relationship between these parameters and body mass is consistent with the large volume of literature on metabolic scaling in fishes [42]–[23], [43]–[45]. However, the slopes of the relationships in the CT_Max_ study (range −0.20 to −0.32; *b* values 0.68 to 0.80) (Fig. 4) are less than have typically been reported in the literature (−0.15–0.20; *b* values 0.80–0.85) [42], [43], [45] and were reported for the cunner at 9°C in the metabolic depression study (*b* = 0.84; Fig. 2). For the RMR of cod, this was likely due to the fact that the measurements for YOY fish were higher than typically reported (see above). This is only one possibility, however, and the fact that the slope for log MMR vs. log body mass was also less than −0.2 (i.e. *b*<0.80) suggests that there are other explanations. These include differences in the methodology, fish condition, and varying environmental factors during rearing (e.g. see [46]). Indeed, scaling exponents for the relationship between log RMR and log body mass range from 0.65 to 0.89 for Atlantic cod [43], [44], [47], [48], and between 1.1 to 0.4 when the data for 89 teleost species of varying life history, lifestyle and metabolic capacity are examined [23].

In contrast to the other metabolic parameters (see Fig. 5A–C), the slope of the relationship between FMS and body mass was essentially zero; i.e. there was no body mass - FMS relationship. This differs from the results of [45] for the ocean pout (*Macrozoarces americanus*), sculpin (*Myoxocephalus scorpius*) and lumpfish (*Cyclopterus lumpus*) where FMS was approximately 1.5 for fish <1 g and 2.3–4.5 for fish>100 g. However, the relationship between FMS and body mass has been described in very few species, and there may be significant intra-specific differences in this parameter as there are for RMR [23]. For example, all the species used in the study of [45] were benthic species, or species of limited swimming capacity, whereas FMS may be more constant for the cod, an active pelagic/schooling species. Further, [49] reported that FMS remained almost constant with body mass in the crucian carp (*Carassius auratus*). Interestingly, this species is also well known for its capacity to metabolically depress in the winter [4], [8].

### Summary

The results of this study provide novel insights into the biology/physiology of cunner, into how the ability to metabolically depress affects the metabolic capacity and thermal tolerance of fishes, and add significantly to our understanding of the plasticity of metabolic function in fishes. For example, in addition to improving hypoxia tolerance [12], it appears that the biochemical and physiological adaptations associated with metabolic depression allow for an enhanced aerobic scope and considerable resilience when exposed to elevated temperatures. Further, they support the majority of studies which show that fish thermal tolerance varies little with body mass [38]–[41], and reveal subtle, but important, differences in how the temperature at which metabolic depression is initiated and the extent of metabolic depression vary with ontogeny.
